# Analysis of Microstructure and Properties of a Ti–AlN Composite Produced by Selective Laser Melting

**DOI:** 10.3390/ma13102218

**Published:** 2020-05-12

**Authors:** Ryszard Sitek, Maciej Szustecki, Lukasz Zrodowski, Bartlomiej Wysocki, Jakub Jaroszewicz, Paweł Wisniewski, Jaroslaw Mizera

**Affiliations:** 1 Faculty of Materials Science and Engineering, Warsaw University of Technology Woloska 141 Str., 02-507 Warsaw, Poland; mszust@gmail.com (M.S.); lukasz.zrodowski@gmail.com (L.Z.); jakub.jaroszewicz@pw.edu.pl (J.J.); pawel.wisniewski@pw.edu.pl (P.W.); jaroslaw.mizera@pw.edu.pl (J.M.); 2 Center of Digital Science and Technology, Cardinal Stefan Wyszynski University, Woycickiego 1/3, 01-938 Warsaw, Poland; bartlomiej.wysocki@pw.edu.pl; 3 MaterialsCare LCC, Zwierzyniecka 10/1, 15-333 Bialystok, Poland

**Keywords:** Ti–AlN composite, microstructure, SEM, Selective Laser Melting (SLM)

## Abstract

Selective Laser Melting (SLM) is a manufacturing technique that is currently used for the production of functional parts that are difficult to form by the traditional methods such as casting or CNC (Computer Numerical Control) cutting from a wide range of metallic materials. In our study, a mixture of commercially pure titanium (Ti) and 15% at. aluminum nitride (AlN) was Selective Laser Melted to form three-dimensional objects. The obtained 4 mm edge cubes with an energy density that varied from 70 to 140 J/mm^3^ were examined in terms of their microstructure, chemical and phase composition, porosity, and Vickers microhardness. Scanning Electron Microscopy (SEM) observations of the etched samples showed inhomogeneities in the form of pores and unmelted and partly melted AlN particles in the fine-grained dendritic matrix, which is typical for titanium nitrides and titanium aluminum nitrides. The AlN particles remained unmelted in samples, but no porosity was observed in the interface area between them and the dendritic matrix. Additionally, samples fabricated with the presintering step had zones with different sizes of dendrites, suggesting a differing chemical composition of the matrix and the possibility of the formation of the phases forming an Ti–Al–N ternary system. The chemical composition in the microareas of the samples was determined using Energy Dispersive X-Ray Spectroscopy (EDS) and revealed differences in the homogeneity of the samples depending on the SLM process parameters and the additional presintering step. The phase composition, examined using X-ray Diffraction analysis (XRD), showed that samples were formed from Ti, TiN, and AlN phases. Porosity tests carried out using a computer microtomography revealed porosities in a range from 7% to 17.5%. The formed material was characterized by a relatively high hardness exceeding 700 HV_0.2_ over the entire cross-section, which depended on the manufacturing conditions.

## 1. Introduction

Additive Manufacturing (AM) methods, which enable manufacturers to obtain complex geometries of manufactured components, are becoming increasingly popular in various industries [1]. Economic and ecological aspects are advantages of the AM methods, because they minimize the necessity of using finishing treatments and reduce material loss. The loose powder left after the process can be reused, and the weight of the used powder is generally almost equal to the weight of the final products. One of the most significant challenges for the material engineering field in this area is the optimization of the AM process manufacturing parameters, which is necessary for the fabrication from new metallic materials and metal–ceramic composites. Titanium was one of the first materials to be processed using the SLM (Selective Laser Melting) technique, and it is widely used in the aerospace and medical industries. Titanium and its alloys are popular in medicine due to their high strength-to-weight ratio, good corrosion resistance, and high biocompatibility with body fluids. Titanium often replaces steels and Inconel alloys in the automotive and aviation industries, because it reduces the mass of the parts while maintaining adequate structural strength. It is also used in bioengineering to produce dental or bone implants [2]. Despite the advantages of titanium, it is difficult to machine due to its low thermal conductivity and high plasticity. The plasticity of titanium and its alloys often causes the fusion of the processed component to the machine tool. Casting titanium alloys is also difficult due to their high melting point, which for pure titanium is 1668 °C [3]. The properties of titanium mentioned above make conventional fabrication methods unfeasible, and it is reasonable to search for titanium processing alternatives in AM methods.

SLM, which is one of the most popular AM methods, makes it possible to obtain Ti6Al4V alloy with up to 99.9% material theoretical density using relevant process parameters [4]. Moreover, parts manufactured using the SLM technique from Inconel 718 could have the same—or even better—mechanical properties as the conventionally obtained material [5]. The SLM technique also allows us to manufacture new alloys and composites made of a mix of powders with different chemical compositions. Chlebus et al. [6] showed that titanium can be alloyed with rhenium with a dissolution of 85%–90% of rhenium particles, maintaining a material with 99.9% theoretical density, despite rhenium having a melting point that is twice as high as titanium. Through SLM technology, Gu et al. [7] manufactured a titanium-based nanocomposite of titanium carbide, improving its mechanical properties. Furthermore, SLM can be used for in-situ synthesis for binary Ti–26Nb alloys, as described by Fischer et al. [8].

The present study aimed to examine the possibilities of manufacturing a new composite, “titanium-based aluminum nitride”, using the SLM technique. Aluminum nitride (AlN) is a ceramic material that is used mainly in electronics due to its semiconductor properties. It is also characterized by high hardness (over 10 GPa) and very good resistance to corrosion [9]. This material oxidizes only at temperatures exceeding 700 °C, but, at the same time, a protective layer of aluminum oxide is created, which prevents it from further oxidization up to 1370 °C. Aluminum nitride melts at 2200 °C followed by decomposition [10]. It is also characterized by a high thermal conductivity that is ten times higher than that of titanium.

To the best of the authors’ knowledge, this is the first article on a Ti–AlN composite manufactured by the SLM method. Although there are reported TiN and AlN materials fabricated by SLM using Ti and Al powders consolidated under N_2_ atmosphere [11], there are no reports concerning the fabrication of three-dimensional Ti–AlN composites. It is expected that aluminum nitride, which is in a much harder phase, may improve the functional properties of commercially pure titanium. It is also possible that in situ synthesis may lead to the formation of new phases of the Ti–Al–N ternary system. There are several reports showing improved functional properties of materials based on Ti_2_AlN or TiAlN phases, which are widely used as thin films [12,13,14,15].

## 2. Materials and Methods

### 2.1. Powder Preparation and Samples Fabrication

The input powder containing 85% Ti Grade 2 (TLS Technik GmbH & Co. Spezialpulver KG, Bitterfeld-Wolfen, Germany) and 15% AlN (H.C. Starck GmbH, Goslar, Germany) (atomic ratios) was prepared by the mechanical mixing of aluminum nitride and titanium in a rotary mill. Samples in the form of 4 mm edge cubes were manufactured by means of the Selective Laser Melting (SLM) method using a Realizer SLM50 (Realizer GmbH, Borchen, Germany) device fitted with Nd:YAG 120W laser with 80 µm focal point diameter. Samples were manufactured in a single process with different scanning speeds and energy densities delivered to the fabricated materials. Sample P1 was made at an energy density of 100 J/mm^3^ and scanning speed of 125 mm/s, while sample P2 was formed at 140 J/mm^3^ and scanning speed of 90 mm/s. Additionally, sample P3 was presintered in a first melting at an energy density of 70 J/mm^3^ and processed in a second melting at an energy density of 100 J/mm^3^. The scanning speed for sample P3 was 90 mm/s and 125 mm/s for presintering and melting stages, respectively. The powder layer was set to 25 µm, hatch distance to 100 µm, and laser power to 15 W at the sintering stage (only for P3) and 30 W at the melting stage (for all samples). The power density for melting stages was directly related to the laser beam scanning speed. The direction of the laser beam movement was changed by 90° with every layer, and scanning was bidirectional (alternating). The parameters used for sample fabrication are summarized in Table 1.

### 2.2. Powder and Sample Characterization

The particle size distributions of the initial powders were analyzed using laser diffraction on the HORIBA LA-950 (Kyoto, Japan) device. The powders were also analyzed in terms of the shape of the particles under a HITACHI TM 1000 (Tokyo, Japan) scanning electron microscope. The phase analysis of the input powder was carried out by X-ray diffraction (XRD) on a Rigaku MiniFlex II (Tokyo, Japan) device equipped with a copper anode (Cu K-α), within an angle range of 2θ 20°–80° and goniometer step of 0.1°, an accelerating voltage of 30 kV, and electric current of 15 mA.

Metallographic samples were prepared using 600 and 1200 grade sandpapers and polished with Al_2_O_3_ suspension. The sample microstructure was observed after etching with Kroll’s reagent, consisting of nitric and hydrofluoric acid diluted in distilled water. The microstructure observations were performed using a HITACHI TM-1000 SEM and HITACHI SU 8000 (Hitachi Ltd., Tokyo, Japan) equipped with Thermo Noran Energy-Dispersed Spectroscopy (EDS) The XRD tests were performed using a Bruker D8 ADVANCE device (Karlsruhe, Germany) equipped with a copper anode at the accelerating voltage of 40 kV and an electric current of 40 mA. Sample porosity tests were carried out using a (Carl Zeiss, XRadia Micro XCT-400) (Oberkochen, Germany) computer microtomography fitted with a Cu (0.5 mm) filter, at the accelerating voltage of 150 kV and pixel size of 15 μm within the angle range of 0°–180°. One thousand radiographs were produced and reconstructed using the XM Reconstructor.

(Carl Zeiss, Germany) program, whereby over 400 virtual cross-sections for each tested sample were obtained. The porosity of the produced samples was determined by a quantitative analysis of a 3D image, using the Bruker CTAn (Germany) program. The region of interest was defined as 1.4 × 1.3 × 1.2 mm. Computer microtomography was chosen instead of Archimedes’ method of calculating density because of external porosity. Microhardness was tested using the Vickers method on a Zwick/Roell ZHV30 (Zwick GmbH & Co. KG, Ulm, Germany) device, under the load of 200 g. Six indentations were made on each sample, and the mean hardness was calculated.

## 3. Results and Discussion

### 3.1. Analysis of Mixed Powders

**Figure 1:**
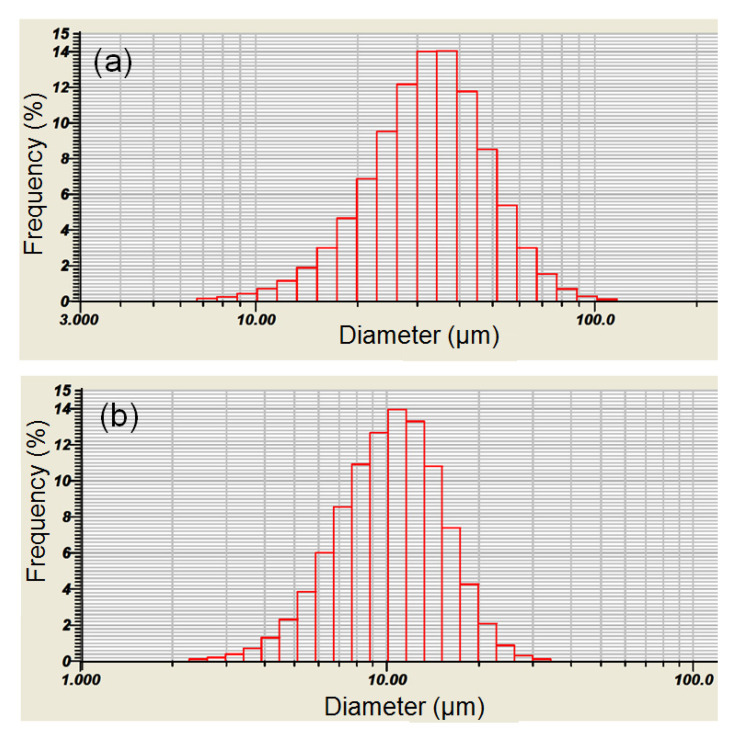
Histograms of Ti–(**a**) and AlN–(**b**) powders’ particle size distribution.

**Figure 2:**
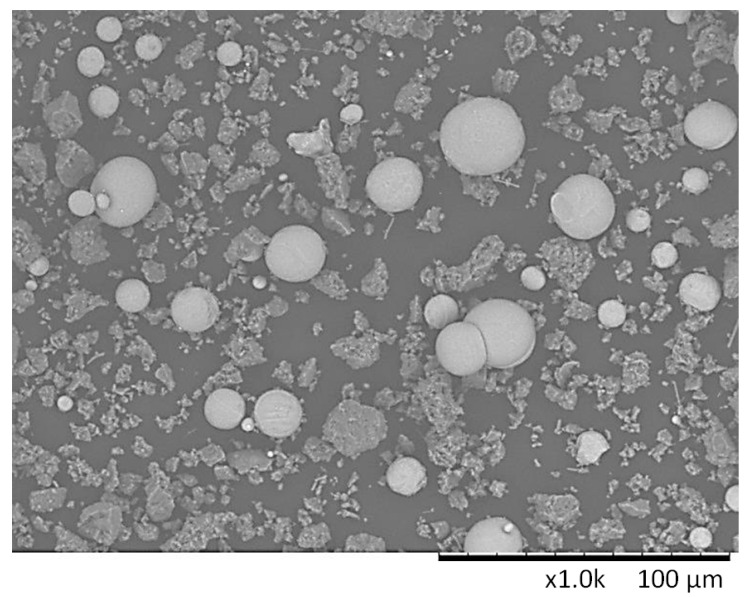
Scanning Electron Microscope (SEM) image of the Ti and AlN powder mixture.

The particle size distribution of Ti and AlN powders is presented on histograms in Figure 1a,b. The histograms of the particle size distribution for both Ti and AlN show a wide range of sizes, which was confirmed by the observations under the SEM microscope (Figure 2).

**Figure 3:**
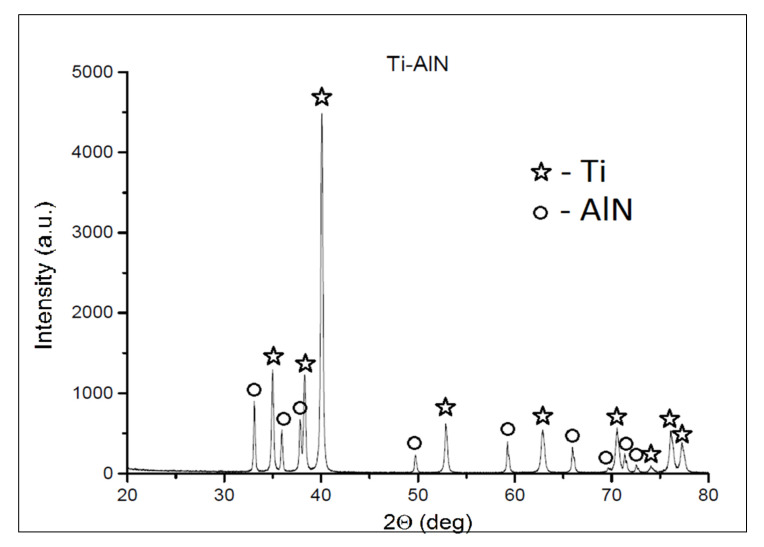
Diffraction pattern of the Ti and AlN powder mixture.

The mean particle sizes were 34.65 µm and 10.96 µm for Ti and AlN powder, respectively. Among the spherical particles of the titanium powder, irregular, much finer, and often agglomerating particles of aluminum nitride were observed. It can be also seen that some of the AlN powder particles were pressed into the Ti powder particles (Figure 2). However, the X-ray diffraction confirmed that mechanical synthesis did not occur during the mixing process and new phases were not created (Figure 3).

#### 3.1.1. Microstructure Characterization of the Samples

**Figure 4:**
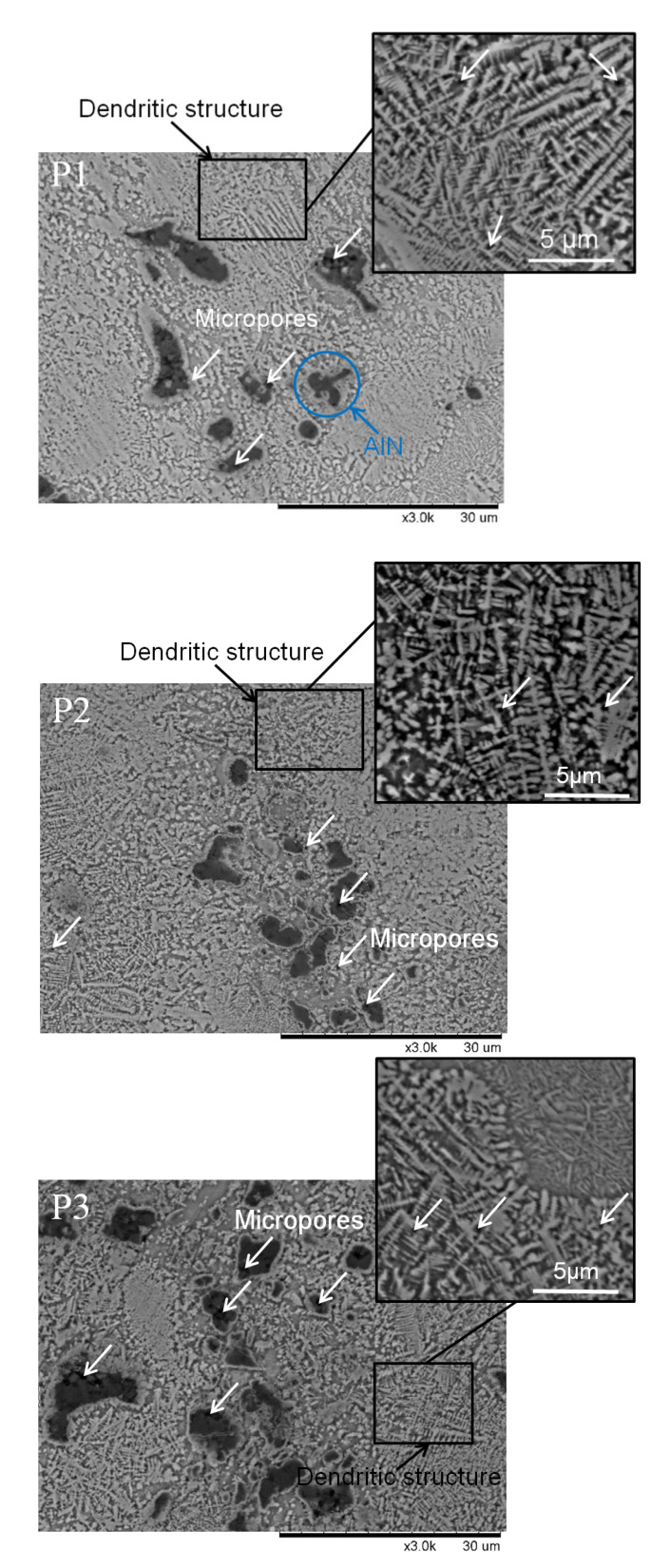
Microstructures of **P1**, **P2,** and **P3** samples etched with Kroll’s reagent. (Micrographs from Scanning Electron Microscope (SEM) in the backscattered electrons (BSE) mode).

The microstructures of all three manufactured samples are shown in Figure 4. Sample etching using Kroll’s reagent showed the fine structures characteristic of the SLM technique, regardless of the energy density used for fabrication. Additionally, inhomogeneity in the form of pores and unmelted and partly melted AlN particles could be observed. A dendritic matrix, which is atypical for titanium fabricated by SLM methods [16,17], was seen in all fabricated samples. The dendrite structure obtained in all fabricated samples is typical for titanium nitrides (TiN and Ti_2_N) and titanium–aluminium nitride (Ti_2_AlN) formed by laser processing [18]. No porosity or cracks were observed in the interface between the AlN particles and Ti matrix. These could have occurred due to the different thermal expansion of Ti and AlN, which could lead to high internal stress after cooling. Sample P3 showed differences in the appearance of the microstructure after etching. There were visible less-etched regions with finer dendritic microstructures than in the P1 and P2 samples. The smaller and less-etched dendritic microstructures suggest a different chemical composition than in the rest of the sample and the possibility of the formation of phases of the Ti–Al–N system, such as titanium–aluminum nitride (Ti_2_AlN) [18].

**Figure 5:**
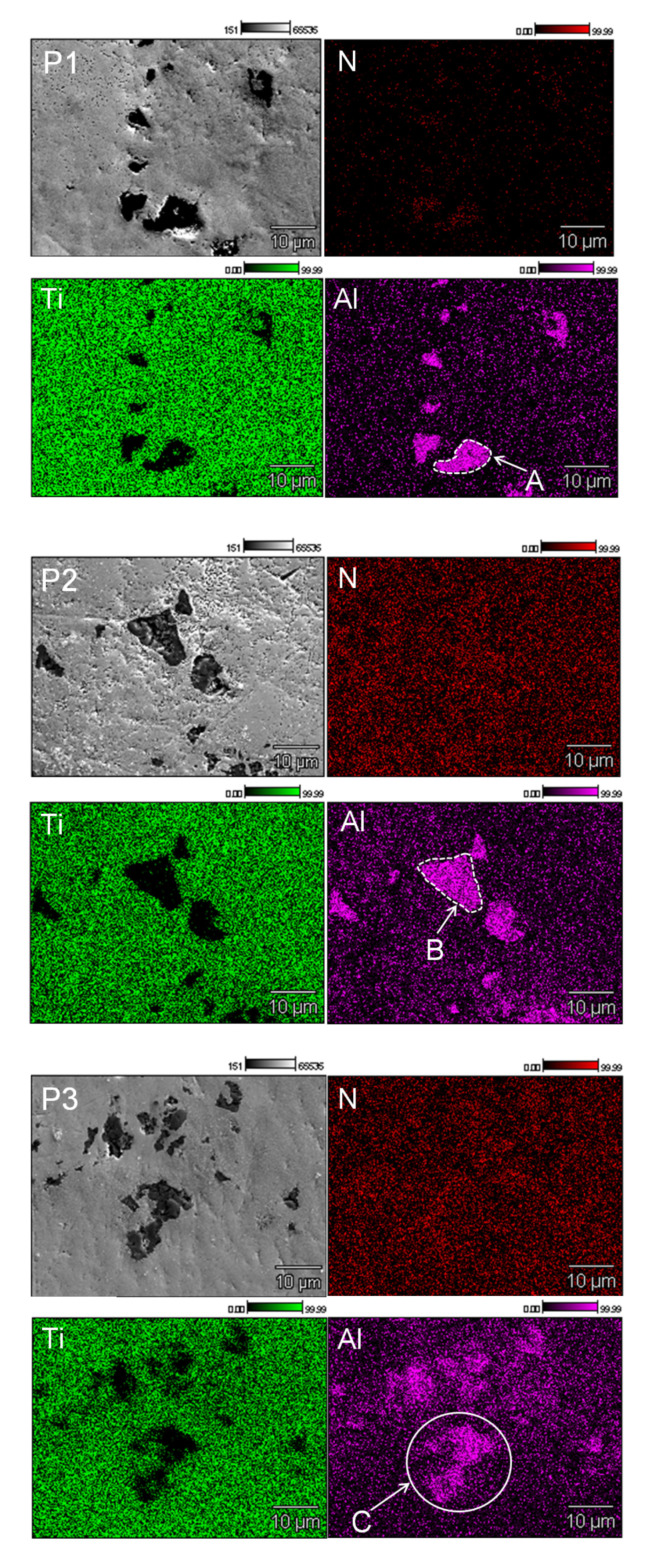
Distribution of elements: Al, Ti, Cr, and N on a cross-section of the samples (Energy-Dispersive Spectroscopy (EDS) mapping). (**A**,**B**) Clear, (**C**)-fuzzy; boundary between AlN and Ti matrix.

EDS maps indicated the presence of aluminum and unmelted AlN particles in the titanium matrix for all fabricated samples (Figure 5). This suggests that only part of the aluminum nitride was dissolved. The most homogenous microstructure was obtained in sample P3, which may indicate that, during the initial sintering, ternary phases from the Ti–Al–N system were created. It can be seen that samples P1 and P2 showed a clear boundary beetwen the AlN particles and Ti matrix (Figure 5A,B), while for sample P3, the boundary between the AlN and Ti matrix was more diffused (Figure 5C). More diffused, fuzzy boundaries between AlN particles and Ti matrix could be associated with the formation of Ti–Al–N ternary phases.

**Figure 6:**
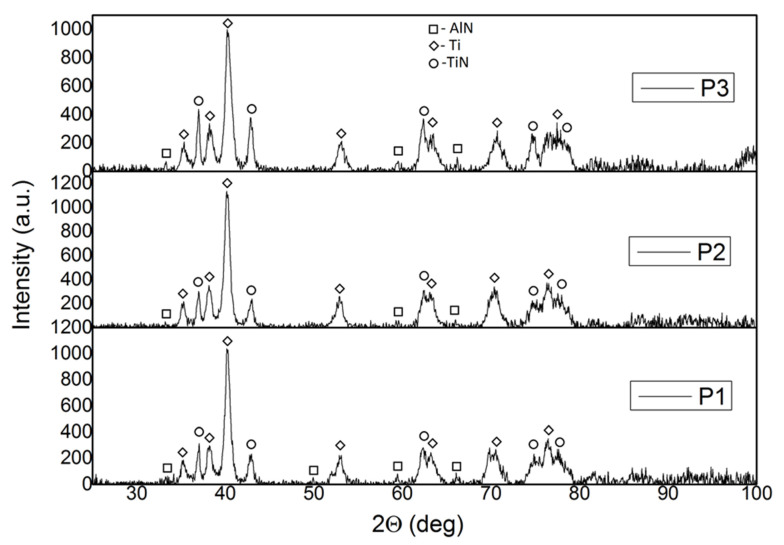
Diffraction patterns of **P1**, **P2,** and **P3** samples produced by the Selective Laser Melting (SLM) technique.

The X-ray analysis data of the manufactured composites using the AM technique are shown in Figure 6. The composites are composed of Ti with a hexagonal structure that fits the PDF (Powder Diffraction File) card 04-002-5384. The aluminum nitride (AlN) has a near-hexagonal structure that fits the PDF card 01-075-1620, and TiN has a near-hexagonal structure that fits the PDF card 04-008-9455.

#### 3.1.2. Analysis of the Samples’ Porosity

**Figure 7:**
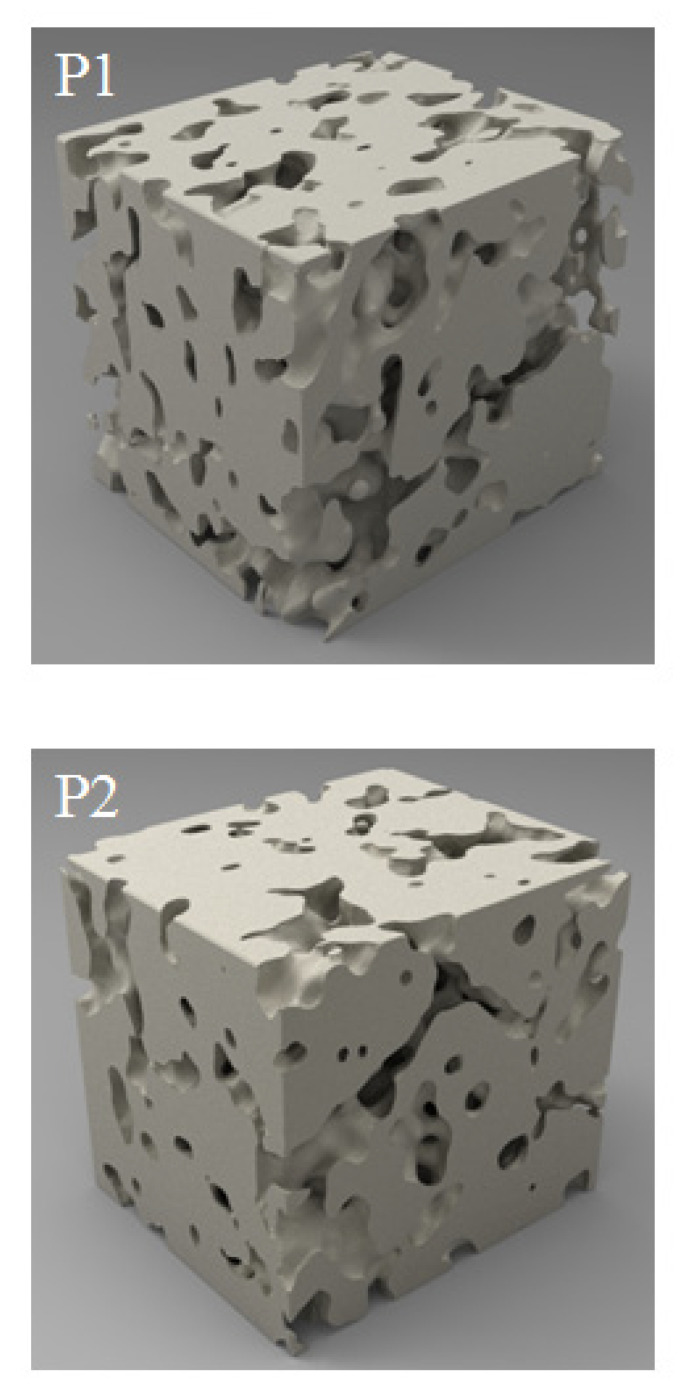
P1 and P2 sample reconstruction based on computer microtomography. The visible 1.4 × 1.3 × 1.2 mm region comes from the inside of samples.

The samples had considerable porosity. On the basis of images made by computer microtomography, 3D sample models were reconstructed (Figure 7). The visible 1.4 × 1.3 × 1.2 mm region comes from the inside of the samples. Increasing the energy density supplied to the sample from 100.0 to 140.0 J/mm^3^ caused a decrease of porosity by over 10% (Table 1).

The mean pore size was in the range 62 to 67 µm, and there was no clear influence of the velocity of the laser beam movement on the porosity of the obtained materials. During the processes, a phenomenon of intense spattering similar to that described by Ly et al. in [19] was observed. This resulted in the pushing away of powder from the melting zone. An initial sintering was carried out for P3 with a lower energy density before melting in order to reduce the influence of this phenomenon on sample porosity. The initial sintering caused minor changes of the porosity for sample P3. For sample P3, decreases from 17.5% to 16.0% and from 67 µm to 65 µm for porosity and pore size, respectively, were observed. The sample porosity was decreased more than two times in sample P2 by increasing the energy density from 100 J/mm^3^ to 140 J/mm^3^. A further increase in energy density might lead to higher porosity, because the higher energy density could affect the chemical decomposition of AlN to the gaseous form of nitrogen that occurs during melting. Even though the currently obtained porosity is relatively high (a minimum of 7% in the case of sample P2), it is a good starting point for further study. The first results concerning the SLM manufacturing of bronzes and titanium in the early 1990s showed theoretical density values of fabricated materials of 85% [20] and 95% [21], respectively. Initially, the porosity in elements manufactured by AM was decreased by Hot Isostatic Pressing (HIP) [22]; however, due to the high costs of this process, AM process parameter optimization [23] and the use of new scanning strategies [24] seem to be more beneficial. It was reported that the porosity of printed objects might increase with their height, which is crucial in terms of their mechanical properties, especially elongation at break [17]. An increase of external porosity is also observed in objects significantly bigger than the samples used for parameter optimization [16]. A decrease in the porosity is essential in order to perform the mechanical testing, which is necessary for engineering applications. One of the easiest possibilities to decrease the porosity in Ti–AlN composites might be use of a machine that heats the working platform to a temperature allowing liquid phase sintering, which, in our study, was only 200 °C. Another solution might be to increase the energy density together with increasing the spot diameter or to search for specific scanning strategies, i.e., double scanning with alternating directions and a lower energy density for the second scan, which could fill the pores produced during the first scan.

### 3.2. Influence of Manufacturing Process Parameters on Microhardness

On the basis of the microhardness measurements, it was found that sample P3, in which presintering was applied, had the highest microhardness. The microhardness of the sample manufactured under the same parameters but without presintering (sample P1) had a lower microhardness. The lowest hardness was found in sample P2, which was made with the highest energy density. This may indicate that relatively high laser energy densities cause a dissolution of aluminum nitride and formation of a less harder phases from Ti-Al-N ternary system.

**Figure 8:**
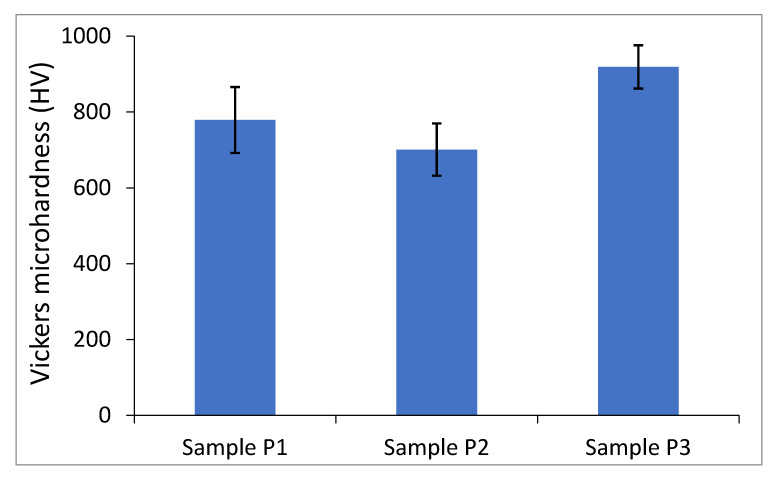
Comparison of the Vickers microhardness HV_0.2_ of the samples: P1 (100 J/mm^3^), P2 (140 J/mm^3^), and P3 (70 J/mm^3^ + 100 J/mm^3^).

The material was characterized by considerable inhomogeneity, which was confirmed by microhardness tests. However, the lowest measured microhardness was 701 HV_0.2_ for sample P2. Diagonals of all impressions were within the range of 25 µm, and therefore they are comparable with the size of Ti powder particles. Comparing these data with the hardness of pure titanium manufactured using the same technology [17], we can conclude that pure titanium with a hardness up to 250 HV_0.2_ was not present in our composite. Our composites had a hardness higher than for TiAl alloy, even after thermal processing [25], but this was considerably lower than the hardness of aluminum nitride itself. A maximum microhardness of 919 HV_0.2_ was obtained in sample P3. It should be noted that the microhardness of the Ti matrix was measured. The unmelted AlN particles exhibited a much higher microhardness, which is beneficial for future applications. The summarized microhardness results of all samples are given in Figure 8.

## 4. Conclusions

On the basis of an analysis of our results, the following conclusions can be made:

(1) The Selective Laser Melting method can be successfully applied to obtain a Ti–AlN composite.
(2) The manufactured composites are characterized by considerable porosity and inhomogeneity (there are areas with a dendritic structure, and axial and nonmolten particles of aluminum nitride).
(3) Increasing the density of energy supplied to the sample from 100.0 J/mm^3^ to 140.0 J/mm^3^ causes a decreased porosity of composite of about 10% and also reduces the hardness.
(4) The work carried out is pioneering and constitutes an introduction to the discussion of the possibility of the manufacture and application of new composite materials from a Ti–Al–N system by using a Selective Laser Melting method.

## Figures and Tables

**Table 1:** Porosity measurements by means of computer microtomography. SD is the value of the standard deviation of measurements.

Sample No.	Scanning Strategy	Energy Density [J/mm^3^]	Scanning Speed [mm/s]	Porosity [%]	Mean Pore Size [µm]
**P1**	Alternating	100.00	125.00	17.5%	67 (SD 25)
**P2**	Alternating	140.00	90.00	7.0%	62 (SD 22)
**P3**	(1) Initial sintering followed by (2) alternating	(1) 70.00 (2) 100.00	(1) 90.00 (2) 125.00	16.0%	65 (SD 24)

The laser power was 15 W and 30 W for the initial sintering and alternating melting, respectively.
